# Plant Flavonoids in Mediterranean Species: A Focus on Flavonols as Protective Metabolites under Climate Stress

**DOI:** 10.3390/plants11020172

**Published:** 2022-01-10

**Authors:** Justine Laoué, Catherine Fernandez, Elena Ormeño

**Affiliations:** Aix Marseille University, Avignon University, CNRS, IRD, IMBE, 13003 Marseille, France; catherine.fernandez@imbe.fr

**Keywords:** biological function, secondary metabolism, biosynthesis, localization, stress response, defense mechanism, antioxidant, leaves, reactive oxygen species

## Abstract

Flavonoids are specialized metabolites largely widespread in plants where they play numerous roles including defense and signaling under stress conditions. These compounds encompass several chemical subgroups such as flavonols which are one the most represented classes. The most studied flavonols are kaempferol, quercetin and myricetin to which research attributes antioxidative properties and a potential role in UV-defense through UV-screening mechanisms making them critical for plant adaptation to climate change. Despite the great interest in flavonol functions in the last decades, some functional aspects remain under debate. This review summarizes the importance of flavonoids in plant defense against climate stressors and as signal molecules with a focus on flavonols in Mediterranean plant species. The review emphasizes the relationship between flavonol location (at the organ, tissue and cellular scales) and their function as defense metabolites against climate-related stresses. It also provides evidence that biosynthesis of flavonols, or flavonoids as a whole, could be a crucial process allowing plants to adapt to climate change, especially in the Mediterranean area which is considered as one of the most sensitive regions to climate change over the globe.

## 1. Introduction

Plants possess thousands of plant secondary or specialized metabolites (PSMs) whose chemical diversity is species-specific. These chemicals play crucial ecophysiological roles as they are implicated in plant interactions with its environment and plant defense against environmental stress conditions [1]. Most of them can be classified within five chemical families: terpenes, phenolic compounds, benzenoids, fatty acid derivatives and alkaloids. Based on their chemical structure, phenolic compounds can be divided into different subgroups including simple phenolics (one phenol unit alone) and polyphenols (with more than one phenolic unit) comprising phenolic acids, flavonoids, tannins, coumarins, lignans, quinones, stilbenes, and curcuminoids [2]. Flavonoids, a major class of polyphenols, widely present in the plant kingdom, represent a large group of PSMs which are either produced constitutively or induced by environmental stresses [3].

The role of flavonoids in protection against UV and drought stress in Mediterranean endemic species has been suggested by recent studies which show a monthly variation in the polyphenol concentrations with the highest levels occurring in summer at midday when drought, temperature and UV radiations are the highest [4,5]. It is also known that geographical factors such as latitude and altitude influence the composition of defensive chemicals including phenolic compound levels [6,7]. Mediterranean species display contrasting polyphenol compositions and concentrations [8,9,10,11] which strongly respond to abiotic stresses. This suggests a key role of phenolic compounds and species-specific functions in plants living in constraining environments such as the Mediterranean region [12]. Accordingly, accumulation of flavonoid concentrations in response to drought have also been reported in some Mediterranean species [13,14,15]. In this context, it is particularly pertinent to understand the role of flavonoids in the adaptation of Mediterranean plant species to rapid climate change.

Flavonoids feature a 15-carbon atom basic skeleton, arranged in the form C6-C3-C6 and present two aromatic rings (A and B) linked by a unit of three carbon atoms (C ring), which may or may not give rise to a third aromatic ring [16]. There are several classes of flavonoids including chalcones, aurones, flavanonols, flavones, isoflavones, flavanols, flavonols, anthocyanins, proanthocyanidins and leucoanthocyanidins. Flavonoids can occur as aglycones, glycosides, and methylated derivatives. More than 6000 different flavonoids have been identified [17] which differ in the number and position of the hydroxyl groups, and their extent of alkylation and/or glycosylation [18]. For example, the flavonols (e.g., quercetin and kaempferol), have a 3-hydroxy pyran-4-one group on the C ring whereas the flavanols catechins have only a 3-hydoxyl group on the C ring [19].

Flavonoids feature a broad spectrum of biological activities in plants which has been related to their chemical diversity and can be linked to their localization within leaf tissues as addressed later in this review. They participate in many cellular activities such as signalling, auxin transport and pigmentation [20,21,22,23]. One of the most highlighted functions of flavonoids is plant protection against abiotic (e.g., drought, salinity, UV radiation and heat) and biotic (e.g., insects and pathogens attack) stresses [24,25,26]. Most commonly, investigations have reported that flavonoids play a role in the modulation of reactive oxygen species (ROS) and possess UV-screening mechanisms [8,27,28,29,30] as described later in this review. Their action as antioxidant is based on (i) their direct capacity to scavenge ROS [31], (ii) their role in inhibition of ROS formation by chelating ion of metals [32,33] and (iii) their ability to activate antioxidant enzymes [19,34]. They also act as indirect plant growth regulators as explained in the last section of this review [21,35].

**Figure 1:**
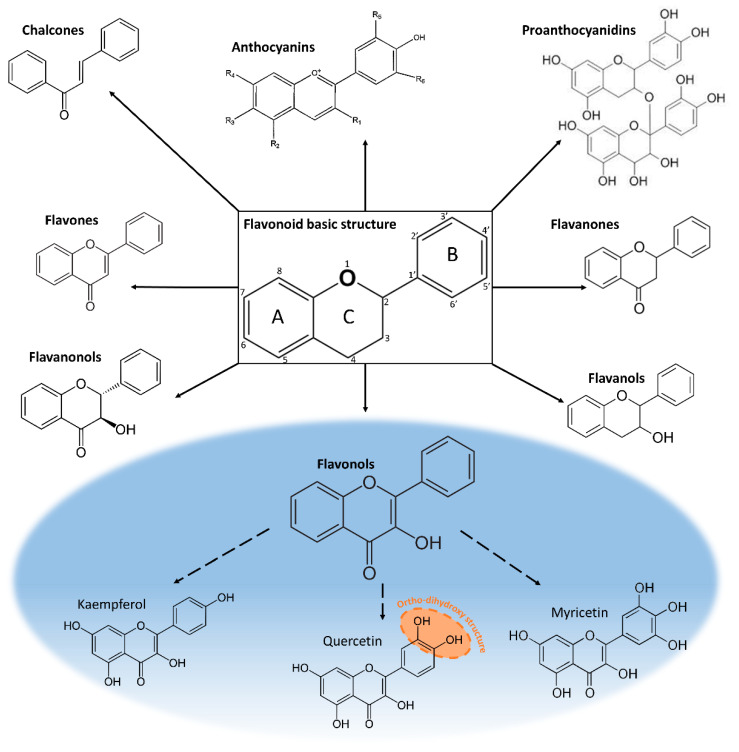
Structure and classification of flavonoids. The main subclasses of major flavonols are circled in blue. The difference between flavonoid groups depends on the chemical structure, the degree of oxidation, and the unsaturation of the linking chain (C3). Flavonols differ from each other in the number and position of the hydroxyl groups (-OH). The *ortho*-dihydroxy structure of quercetin is circled in orange.

One of the most important flavonoid sub-groups are flavonols which include kaempferol, quercetin and myricetin, the most studied flavonols [36]. Numerous functional roles have been attributed to flavonols, especially their role as antioxidant molecules and UV-screening pigments since they have long been considered as the most effective UV-B absorbers thus conferring strong photo-protection [37]. The catechol group in the B-ring (Figure 1), as observed in the flavonol quercetin and its derivates, makes flavonols the most effective antioxidant compounds and therefore key compounds in the plant responses to changing climate. Flavonols seem particularly important in Mediterranean plant species as studies usually report a high concentration of flavonols such as quercetin under excess light and drought stress [8,9,38]. The higher distribution of quercetin in Mediterranean plants may explain their ability to cope with a dry climate and high solar irradiance as this flavonol is an excellent antioxidant [31].

This review resumes the chemical and physical properties, biosynthesis, storage localization, and biological functions of plant flavonoids, and relationships among these five features with a deeper focus on flavonols. We finally focus on the response and protection conferred by flavonols under climate stress in Mediterranean plants species. All these sections are described by reviewing a number of interdisciplinary studies (biochemistry, ecology and ecophysiology studies) that have used genomic, transcriptomic, metabolomic and imaging approaches.

## 2. Chemical and Physical Properties of Flavonols: Relationship with Their Function

Flavonols, and flavonoids as a whole, have historically been studied for their functions in UV-B protection in plants because they display strong absorption in the UV spectral region and exhibit fluorescence with excitation maxima about 350–370 nm [39,40]. Their biological activity in plants remains to be elucidated and some of them are still under debate [37]. Overall, it has been proved that their functions depend on their chemical and physical properties [41].

Flavonols, closely related in structure to flavones, are characterized by the presence of a hydroxyl group (-OH) at the C3 position and a carbonyl function (C=O) at the C4 position, both on the C ring [42] (Figure 1). In the structure of flavonoids, the C ring may be a heterocyclic pyran—which yields flavanols (e.g., catechin) and anthocyanidins, or pyrone—which yields flavonols (e.g., kaempferol, quercetin, myricetin), flavones (e.g., luteolin, apigenin), and flavanones (e.g., naringenin, eriodyctiol) [43] (Figure 1). Flavonol diversity is due to the aglycone structure and its oxidation or reduction state, the position of the hydroxyl group and the degree of hydroxylation [44].

Chemical structure and physical properties of flavonols are intimately linked to their biological functions. Several studies have shown that their antioxidant activity increases with the number of hydroxyl groups and depends on the -OH position within the molecule [45,46] (Table 1). Differences in the chemical structure and the relationship to their antioxidant activities are summarized in Table 1. The substitution patterns in kaempferol, quercetin and myricetin are 3,5,7,4′-OH, 3,5,7,3′,4′-OH and 3,5,7,3′,4′,5′-OH, respectively (Figure 1). The presence of a third-OH group in the B ring in myricetin does not enhance the effectiveness against aqueous phase radicals compared to quercetin [46] (Table 1), highlighting the importance of the *ortho*-dihydroxy structure in the B ring of quercetin which allows electron delocalization and thus increases their antioxidant activity [47] (Figure 1).

Similar to other PSMs, flavonols often exist in glycosylated forms (also referred to as glycoside conjugates) which are formed by an aglycone core bound to glycosidic sugars through oxygen or more rarely carbon atoms [49,50]. The glycosidic sugar is generally glucose, but also frequently galactose and rhamnose. The most common flavonol glycosides containing glucose are astragalin (kaempferol 3-*O*-glucoside) and isoquercetin (quercetin 3-*O*-glucoside) which have as aglycone cores kaempferol and quercetin, respectively. Myricetin can be coupled with rhamnose giving myricitrin (myricetin 3-*O*-rhamnoside) [51,52]. Glycosylation of flavonols is of high biological importance since it tends to decrease their antioxidant activity [53,54,55]. For example, Rice-Evans et al. [46] have demonstrated that glycosylation of quercetin blocks the -OH at the C3 position in the C ring thus reducing its antioxidant capacity. Glycosylation also preserves the reactive -OH groups from auto-oxidation [56]. Glycoside forms of flavonols is an important structural modification which influences their physical properties by increasing their solubility, stability and bioavailability [57]. Glycosylation modifies their cellular and tissular repartition too [49] (see chapter “Biosynthesis and storage of flavonols: relationship with their function”). Overall, the functional diversity and biological activities of flavonols are due to the modification of the aglycone core. The attachment of functional groups such as sugars, hydroxyl and methyl groups gives flavonols their ability to have different biological roles and storage sites in the plant.

Most flavonols (as well as flavones) exhibit two major absorption bands: Band I (320–385 nm) own to the B ring absorption, and Band II (250–285 nm) which refers to the A ring absorption. Increase in the numbers of hydroxyl groups in flavonoids induces an adsorption shift towards the red band such as 367 nm in kaempferol, 371 nm in quercetin and 374 nm in myricetin [58] (Table 1). The structure of flavanones leads to different UV absorption wavelengths (e.g., lower maximum absorption wavelength) compared to flavones and flavonols as well as a lowered antioxidant activity (Table 1). These differences can lead to various and different biological functions. In fact, dihydroxy B ring-substituted flavonoids have a greater antioxidant capacity, while their monohydroxy B ring-substituted counterparts have greater ability to absorb UV wavelengths [28].

## 3. Biosynthesis and Storage of Flavonols: Relationship with Their Function

**Figure 2:**
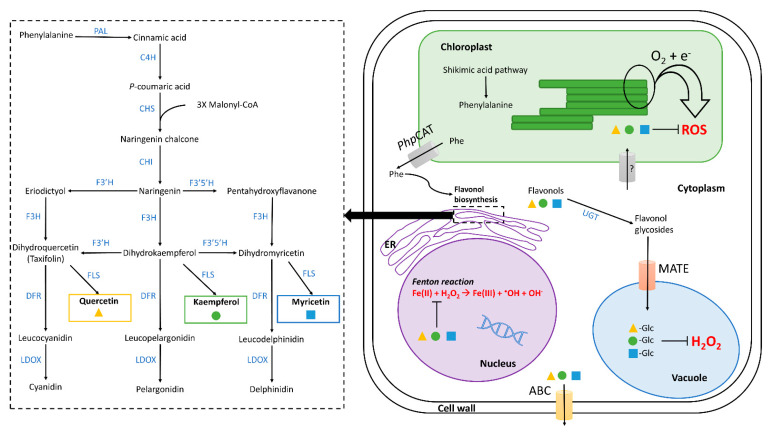
Biosynthesis and subcellular localization of flavonols in leave tissues. Flavonols are synthesized in the cytoplasm, on the cytosolic face of the endoplasmic reticulum (ER) (**right picture**). The different enzymes involved in their biosynthesis (**left box**) are shown in blue and flavonols are indicated and framed by different colors. The pathway shown represents the general pathway found in model plants such as *Arabidopsis thaliana*. The first step begins with the synthesis of phenylalanine in the chloroplasts which is then transported to the cytosol via the phenylalanine plasticial cationic amino acid transporter (PhpCAT), identified in petunia. Abbreviations are as follows: C4H, cinnamate 4-hydroxylase; CHI, chalcone isomerase; CHS, naringenin-chalcone synthase; DFR, bifunctional dihydroflavonol 4-reductase/flavanone 4-reductase; F3H, flavanone 3-hydroxylase; F3′H, flavonoid 3′-hydroxylase; F3′5′H, flavonoid 3′5′-hydroxylase; FLS, flavonol synthase; LDOX, leucoanthocyanidin dioxygenase; PAL, phenylalanine ammonia-lyase; Phe, phenylalanine; UGT, UDP-dependent glucosyl transferase. Once synthetized, flavonols can be subjected to various modifications (glycosylation, methylation, etc.) and be stocked into vacuoles. They are transported into different compartments and through cells by MATE (multidrug and toxic compound extrusion) and ABC (ATP binding cassette) transporters families. In nucleus, vacuole, and chloroplast, flavonols will inhibit ROS accumulation.

The precursor of flavonoids is the amino acid phenylalanine which is the intermediate in the biosynthetic transformations leading from shikimic acid to phenylpropanoids [59] (Figure 2). Phenylalanine, derived from the shikimate pathway, is synthetized in chloroplasts and then transported into cytosol by a phenylalanine plastidial cationic amino acid transporter (PhpCAT) identified in *petunia* flowers by Widhalm et al. [60] (Figure 2) [61]. Flavonoids are synthesized through the phenylpropanoid pathway with a step transforming the phenylalanine into cinnamic acid and then into *p*-coumaric acid (Figure 2). These two compounds are synthesized via the phenylalanine ammonia-lyase (PAL) and cinnamate 4-hydroxylase (C4H), respectively. The first enzyme involved is the chalcone synthase (CHS) producing naringenin chalcone through p-coumaric acid and three malonyl-CoA. Then chalcone isomerase (CHI) leads to naringenin from which all flavonoids derive. Flavanonols (or dihydroflavonols) arise from flavanones by the intervention of flavanone 3-hydrolxylase (F3H) a key enzyme in the flavonoid pathway. F3H catalyzes the oxidation of naringenin into dihydrokaempferol (colorless dihydroflavonol) that subsequently can be hydroxylated on the 3′ or 5′ position of the B ring, by flavonoid 3′-hydroxylase (F3′H) or flavonoid 3′,5′-hydroxylase (F3′5′H), producing dihydroquercetin (taxifolin) and dihydromyricetin, respectively. In addition, naringenin may be directly hydroxylated by F3′H or F3′5′H to give, respectively, eriodictyol and pentahydroxy-flavanone, which are again hydroxylated by F3H into dihydroquercetin (taxifolin) and dihydromyricetin [62] (Figure 2).

A set of enzymes involved in flavonol biosynthesis (CHS, CHI, F3H, FLS, and F3′H) has been detected (for a review see Ferrer et al. [36]). Their biosynthesis is triggered in response to light and ROS stress occurring in photosystems. The three main flavonols—quercetin, kaempferol and myricetin—are formed from dihydroflavonols by the action of flavonol synthase (FLS). FLS is the most important enzyme in the biosynthesis of flavonols and it is in competition with dihydroflavonol 4-reductase (DFR) involved in the synthesis of leucoanthocyanidins [63] (Figure 2). The basic structure of flavonols (see Figure 1) is obtained by oxidation of dihydroflavonols by FLS [64].

Flavonoid biosynthesis is first regulated at genetic and transcriptional levels [65]. Such regulations have been described in various model plant species thanks to the availability of many mutants affecting the expression of several flavonoid biosynthetic genes [66,67]. MYB (myeloblastosis) and basic helix–loop–helix (bHLH) transcription factors, together with WD40 proteins are the main transcriptional regulators of the flavonoid biosynthetic pathway genes [68,69]. Flavonol biosynthesis is more specifically regulated by different MYBs transcription factors as identified in *Arabidopsis thaliana*, *Prunus persica* and grapevine [70,71,72,73]. In *Arabidopsis*, AtMYB11, AtMYB12, and AtMYB111 from the R2R3-MYB gene family activate on their own the CHS, CHI, F3H, and FLS promoters [70]. Interestingly, the authors identified additional genes including UDP-glycosyltransferases (UGTs) and demonstrated that the accumulation of flavonol glycosides correlates with the expression domains of the different MYB factors.

Biosynthesis of flavonoids mainly occurs in the cytoplasm, more precisely in the cytosolic face of the endoplasmic reticulum as shown in many different species [74,75,76] (Figure 2). Furthermore, some of the enzymes involved in flavonol biosynthesis have been shown to co-localize in the nucleus, consistent with the idea that some flavonols are directly or indirectly involved in the protection of DNA against ROS oxidative damages in particular by supressing the Fenton reaction (i.e., sequestration of metal ions Fe(II); Figure 2) [48,77,78]. Flavonols have also been detected in chloroplasts, and a study from Zaprometov and Nikolaeva [79] concluded that chloroplasts are capable of flavonoid biosynthesis but this hypothesis needs to be confirmed by other experimental support [79,80]. The presence of antioxidant flavonols in chloroplasts could be explained because chloroplasts are a major source of ROS. Overall, the presence of flavonols in various cell compartments can be mainly explained by their antioxidant activities (for a review see Hernández et al. [80]), but their subcellular transport needs to be better elucidated.

Once synthesized, the end-products of the flavonoid pathway such as flavonols are transported towards various cellular organelles (i.e., vacuole, chloroplasts and nucleus; Figure 2). Intracellular transport in leaf tissues occurs primarily to the vacuoles of different cells (stomata guard cells, epidermal and subepidermal cells). Their transportation from the biosynthesis site to the storage site is driven by different transporters such as multidrug and toxic compound extrusion (MATE) [81]. In vacuoles, flavonols are mainly stored in glycosylated forms—since glycosylation increases their solubility in the aqueous cellular compartment [82]—where they have been suggested to reduce the H_2_O_2_ concentration [83]. Although vacuoles from epidermal cells are their main flavonol reservoirs, flavonols are also transported and stored in the cell walls of the epidermal cells as methylated flavonol glucosides [84] and within the leaf cuticle [85]. Flavonoids (including flavonols) are also synthesized and then accumulated in glandular trichomes of leaves, with higher concentrations in leaves exposed to high levels of light (as shown in the Mediterranean species *Phillyrea latifolia*; [86]). All these studies illustrate that flavonoids are mainly stored in the outer leaf tissues (epidermis, cuticle and outer storage structures) which optimizes their role as UV-screeners or ROS scavengers. In addition, quercetin-glycosides have been detected in the mesophyll of leaves subjected to drought stress which is consistent with their potential function as H_2_O_2_ scavengers [38]. Likewise, flavonoids reach the highest concentrations in leaves compared to other plant organs [87], which is related to their protective role under excess light (see Section 4). Imaging techniques, such as confocal microscopy, are used to precisely locate flavonoids within leaf tissue [88].

The transport mechanisms of flavonoids from the biosynthetic site to the storage site (within cellular organelles and tissues) but also between plant organs, remain poorly understood. The transport of flavonoids to the chloroplast is not known. Some hypotheses have been proposed for flavonoid transport including membrane vesicle-mediated transport and membrane transporter-mediated transport [89,90]. Several transporters are known to be involved in flavonoid transport such as MATE (multidrug and toxic compound extrusion) transporters cited above (for transport of flavonoids between the cytosol to the vacuole) [81,91] and the ATP binding cassette (ABC) (Figure 2) [92]. The later participate in long-distance unidirectional transport of flavonoids (e.g., naringenin, dihydrokaempferol and dihydroquercetin) between roots and shoots [93]. The transport of flavonoids between different plant organs seems crucial to respond to the various stresses undergone by the plant.

## 4. Flavonols in Plants: An Important Polyphenol to Cope with Rapid Climate Change

In the context of climate change, the increasing CO_2_ concentration in the atmosphere implies global warming [94] and depletion of the stratospheric ozone layer, resulting in UV-B radiation increases [95]. At the regional scale, the Mediterranean is a critical hot spot in the context of climate change due to the expected increase in surface temperatures, drought episodes and solar radiation exposure [96,97,98]. By the end of the twenty-first century, precipitation will decrease at a rate of around −20 mm/K (or −4%/K) in this region and temperature will warm 20% more than the global average, especially in summer (i.e., 50% larger than global warming) [98]. A warmer climate in the Mediterranean area will also cause variations in the hydrological cycle consisting of a rise in both sea level and soil salinity [99]. Arid regions are the most prone to salinization due to the precipitation scarcity leading to low drainage of salts in soil [100]. Ultraviolet (UV) radiation, drought, warming and salinity expected in the Mediterranean region threaten plant development as they negatively impact physiological and biochemical processes, resulting in reductions in plant growth and reproduction success (fitness) [24]. To counterbalance such climatic stresses, plants develop physical and chemical defenses [101], the later being partly represented by PSMs, including polyphenols [12,102].

To a certain extent, Mediterranean plants are able to tolerate warming, increased drought and UV radiation excess [103]. Morphological and physiological plasticity have been observed in Mediterranean trees, shrubs and herb species (for a review see Matesanz and Valladares [103]). Plasticity differs not only among species and populations but also among traits and environmental factors. Species with high phenotypic plasticity, and in particular those able to evolve rapidly, have an evolutionary advantage, especially under rapid climate change. Among the plasticity traits, plant metabolic plasticity is crucial for resistance and adaptation to various abiotic stresses and has thus been used as an indicator of plant survival in a changing environment. Mediterranean species display very contrasting flavonoid composition and concentration [12], thus suggesting a species-specific response to climate stressors. A study of Sosa et al. [104] highlighted the diversification in composition and content of flavonoids in a same species (*Cistus ladanifer*) from different populations and locations (different climatic conditions). This result suggests that flavonoids could have various ecological functions strongly linked to environmental conditions. All these studies illustrate the fact that the metabolic plasticity can be an important asset for Mediterranean plants to cope with changing climate. In recent decades, various studies have demonstrated the effect of climate-related stresses on flavonoid metabolism. Some of them reveal an enhancement of flavonol production under drought, heat, UV radiation and salinity, as well as their protective role under these stress conditions as described hereafter (Table 2). These studies strongly suggest an important role of flavonols to cope with unfavorable Mediterranean environmental conditions, especially with climate change.

### 4.1. Drought

Flavonol concentrations increase under drought stress as found in several species [118,119,120,121]. A recent study on the model species *Arabidopsis thaliana* has also confirmed by transcriptomic evidence the enhancement of flavonol metabolism under drought conditions [105]. In fact, drought regulates key genes coding for the enzymatic activity involved in flavonol biosynthesis such as chalcone isomerase (CHI), flavonoid 3′-hydroxylase (F3′H), flavanone 3-hydroxylase (F3H) and flavonol synthase (FLS) which results in increased flavonol concentrations [122,123] (Figure 2). Moreover, it has been shown that some transcription factors such as MYB and bHLH families play a role in the accumulation of flavonoids resulting in enhanced drought tolerance [124,125]. It is also now known that there exists a relationship between aglycone/glycone flavonols and the oxidative stress, the glycosylated forms of flavonols being less effective antioxidants [54,55]. Despite their contrasting antioxidant protection, an accumulation of quercetin 3-O-glucosides and a decrease in the antioxidant enzyme activity have been observed under water stress in leaves of the Mediterranean species *Fraxinus ornus*, suggesting that these glycoside forms of flavonols could act as H_2_O_2_ scavengers during water stress [38]. The precise role of flavonoid glycosylation under drought remains complex to evaluate because of the signaling crosstalk between flavonoid production and stress response. Other recent studies also highlight the sensitivity of flavonol concentration in response to drought in different Mediterranean tree species such as *Pinus pinaster* Ait. and *Quercus ilex* L. [14,15,126]. All these studies support the idea that increasing foliar concentration of flavonoids is a key defense strategy under water depletion. They also reveal the strong capacity of Mediterranean species to reinforce the flavonoid metabolism to cope with drought.

### 4.2. Warming

Warming, expected to be paralleled by aggravated drought in the Mediterranean region, is an important stress factor affecting plant growth and survival [127]. In plants, many biochemical reactions are sensitive to temperature, the stress from which varies according to the pic of temperature reached and duration [128]. As a consequence of heat, leaf water status and stomatal conductance are affected, resulting in a higher ROS production [129]. Temperature has a high influence on flavonoid metabolism, especially on anthocyanin accumulation which is reduced in response to high temperatures, as shown in grapevine and apple fruit [130,131,132]. In shrub and a conifer species, recent studies have reported an effect of enhanced temperature which leads to a reduction in flavonoids in stems [133] and leaves [134,135], this being related to an increase in plant growth. In those studies, results support the theory that under non-limiting resources, plants exposed to a moderate elevated temperature will use carbon for growth rather than for synthesis of defense compounds [136].

Concerning Mediterranean species, they have developed a wide range of adaptative traits to survive to summer climate conditions, including both elevated temperatures and drought [137]. For example, several studies have been conducted on the typically Mediterranean species, *Cistus ladanifer* L., whose leaves and stems secrete an exudate rich in secondary metabolites, in particular flavonoids [138,139]. Seasonality, closely related to temperatures, influences strongly flavonols content in *C. ladanifer* L. [139,140]. The maximum secretion of flavonoids in the exudate of this species is produced during summer when plants suffer the most from high temperatures, and also UV irradiation and water stress [140]. Few recent studies reported the effect of warming on the concentration of phenolics, especially when combined with others stress such as drought [113,141]. The study of Zandalinas et al. [113] on *Citrus* plants highlighted that the combination of water stress and heat stress lead to higher levels of flavonols (i.e., kaempferol derivates) than in heat stress only. These findings are particularly interesting as the combination of drought and heat is the most recurrent condition in Mediterranean regions. It has also been reported in a typical Mediterranean tree species (*Quercus ilex* L.) that in winter (at the lowest temperatures), flavonol contents were the highest [112]. The highest amount of flavonols in this season can be explained by their contribution to photoprotection as in winter low photosynthetic activity and low excess energy dissipation can increase the risk of photodamage. Despite all this evidence about the role flavonoids play in Mediterranean plant adaptation to environmental changes, future studies need to address their efficiency when these changes operate rapidly.

### 4.3. UV Radiation

High solar radiation is one of the most important changes plants will have to cope with under future climate change [142]. UV radiation is generally divided into three classes based on the light wavelength: UV-C (<280 nm), UV-B (280–315 nm), and UV-A (315–400 nm) [143]. Flavonols have largely been considered as an important role in UV protection by inhibiting ROS generation, and ROS quenching once they are formed [28,37,115]. In addition, as many other flavonoids, they have the capacity to absorb solar wavelengths in the range between 280 and 320 nm (UV-B) [37]. However, in the recent past, the idea of flavonols as the most effective UV-B absorbers has been questioned [27,115,144]. Due to their tissue distribution, often on leaf surface (e.g., cuticle, trichomes), they could act as UV-screening, but photoprotection is possibly not their most important function [27,109,145]. UV-induced increases in the ratio of dihydroxy to monohydroxy B-ring substituted flavonols glycosides (such as quercetin to kaempferol ratios) as reported in different plant species exposed to various proportions of UV radiation [144,146,147,148]. Indeed, kaempferol and quercetin are both able to absorb light in the UV-A and UV-B regions, but they present variations in their ROS scavenging properties. The dihydroxylated B-ring of quercetin provides increased antioxidant activity relative to the monohydroxylated ring of kaempferol which explains the higher accumulation of quercetin than kaempferol under oxidative stresses [46,149]. In addition, a screening advantage for quercetin glycosides over kaempferol glycosides could be due to the higher capacity of quercetin glycosides to dissipate UV-B excitation energy through tautomerization [40].

A recent study from Hectors et al. [110] highlighted the role of the rhamnosylated kaempferol and quercetin glycosides during UV acclimation in *Arabidopsis thaliana*. The concentration of these compounds increased because of UV-stress and the resulting oxidative stress. However, the biological role of these flavonol derivatives remains unclear as they feature less effective antioxidant activity than their aglycone forms [54,55]. It has thus been suggested that accumulation of flavonol glycosides constitutes a reserve of flavonols which can be used during long-term UV acclimation and does not represent the first line response upon exposure to UV radiation [37]. Studies on Mediterranean plants species usually report a concentration of dihydroxy B-ring-substituted flavonols (quercetin) greater than that of monohydroxy-flavonols (kaempferol) under excess light and drought stress [8,9,38]. The higher concentration of quercetin in Mediterranean plants compared to kaempferol is probably related to their high adaptation to light exposure as quercetin is an excellent antioxidant [31]. Comparatively, the reverse is observed in shaded plants with a decrease in the quercetin: kaempferol ratio [144,150].

### 4.4. Salinity

Soil salinity may occur for two reasons: natural accumulation of salt in the soil over long periods or human-induced accumulation due to activities that change the hydrologic soil balance [151]. In Mediterranean regions, increasing crop irrigation is crucial to cope with the drier climate but it often causes soil salinization because water used for irrigation is groundwater whose high salinization is due to seawater intrusion [152]. In addition, the natural accumulation of salt in the soil can be due to poor precipitation, which can be observed in Mediterranean regions, resulting in lower drainage of soil water. In plants, the excess of salt in soils reduces their ability to uptake water and competes with the mineral nutrient metabolism reducing plant growth [153,154]. Under salt stress, a reduction in photosynthesis is observed due to the reduction in water potential [155,156]. In fact, salt stress can lead to stomatal closure reducing CO_2_ uptake by leaves thereby exposing chloroplast to an excess of energy leading to the generation of ROS and leaf oxidative damage [157]. Salt stress and water deficit stress show a high degree of similarity in physiological, biochemical and molecular responses, probably due to the fact that salt stress brings osmotic effects [158]. As shown in many studies, salinity stress induces flavonol accumulation to mitigate oxidative stress [159,160]. These results are also supported by genomic evidence where key genes of the flavonol pathway were upregulated under salt stress (e.g., FLS, F3′H and F3H) [117,161].

Salt stress’ effect on flavonoid contents in Mediterranean species remains poorly documented. Tattini et al. [162] have described the response of salinity on three different evergreen Mediterranean species (*Olea europaea* L., *Phillyrea latifolia* L. and *Pistacia lentiscus* L.) which are widely distributed in dry coastal areas of the Mediterranean basin, where soil salinity concentrations accumulate especially during the warm summer season. In the two least salt stress-tolerant species (i.e., *O. europaea* and *P. latifolia*), phenylpropanoid metabolism was upregulated compared to the species that better utilizes Na^+^ and Cl^−^ for osmotic adjustment (*P. lentiscus*). The most salinity-sensitive species reduced their growth more than the most resistant species to better devote their energy to antioxidant defenses (i.e., flavonoid biosynthesis).

## 5. Flavonols as Antioxidants: A Unifying Mode of Action against Climate Stresses

All factors previously described (light, high temperatures, water deficit and salinity), trigger ROS production under stress conditions overwhelming the scavenging mechanisms of the antioxidant system in plants and eventually leading to several cellular damages including alteration of DNA which can cause cell death [163]. Major sites of ROS production in plants are located in the chloroplast, mitochondria and peroxisomes [164].

Flavonol biosynthesis is almost exclusively enhanced due to triggered ROS formation associated with oxidative stress [19,28]. The major source of ROS production occurs during the photosynthetic electron transport system. In case of drought stress, partial or total stomatal closure allows plants to reduce evapotranspiration but also limits the entrance of CO_2_ and thus net photosynthesis leading to an excess of unused light energy and perturbation in the chloroplastic electron chain since chloroplasts continue to absorb light energy. As a result, leaf cells produce a significant rise of ROS [165]. An imbalance is created within the photosynthetic reactions because the energy supply (NADPH, ATP) exceeds the demand and electron acceptors become depleted. These electrons are transferred to oxygen, resulting in the production of ROS [166] which include superoxide (O_2_^−^), hydrogen peroxide (H_2_O_2_), hydroxyl radical (^•^OH), singlet oxygen (^1^O_2_) and perhydroxyl radical (HO_2_^•^) [83].

The main explanation for flavonols acting as antioxidant relies on the high reactivity of their hydroxyl substituents as shown in the following reaction [44]:

F-OH + R^•^ → F-O^•^ + RH

Specifically, the B-ring hydroxyl structure is the main driver of flavonoid potential to scavenge ROS [44]. Flavonols are greatly antioxidant because of their ability to donate electrons or hydrogen atoms. They act as antioxidants by several mechanisms. The first on is direct scavenging of ROS, as described in the reaction above. They also inhibit ROS formation through the chelation of metals. For example, quercetin present a strong capacity to chelate ions of metals such as Fe and Cu-ions thus preventing free radical formation including the damaging ROS [32,33]. Due to their specific structure, in particular the hydroxyl groups, flavonols can form metal flavonol complexes [167]. They also can inhibit the enzymes that participate in the generation of free radicals (e.g., glutathione S-transferase and NADH oxidase), or potentially activate some antioxidant enzymes possessing radical scavenging capacity [19,34,168].

To determine the potential role of flavonols in planta, in vitro assays were first performed, showing that flavonols can directly scavenge ROS [31]. However, data supporting their role as ROS scavengers in living plants remain poorly documented (for a review of effective role of flavonoids as antioxidant in plants see Agati et al. [86]). More recently, studies performed under controlled conditions in laboratory on the model plant *Arabidopsis thaliana* and some crop species have correlated the increase in flavonols and the decrease in ROS, suggesting an antioxidant role of flavonols under stress conditions [106,109].

## 6. Flavonols as Indirect Growth Regulators

**Figure 3:**
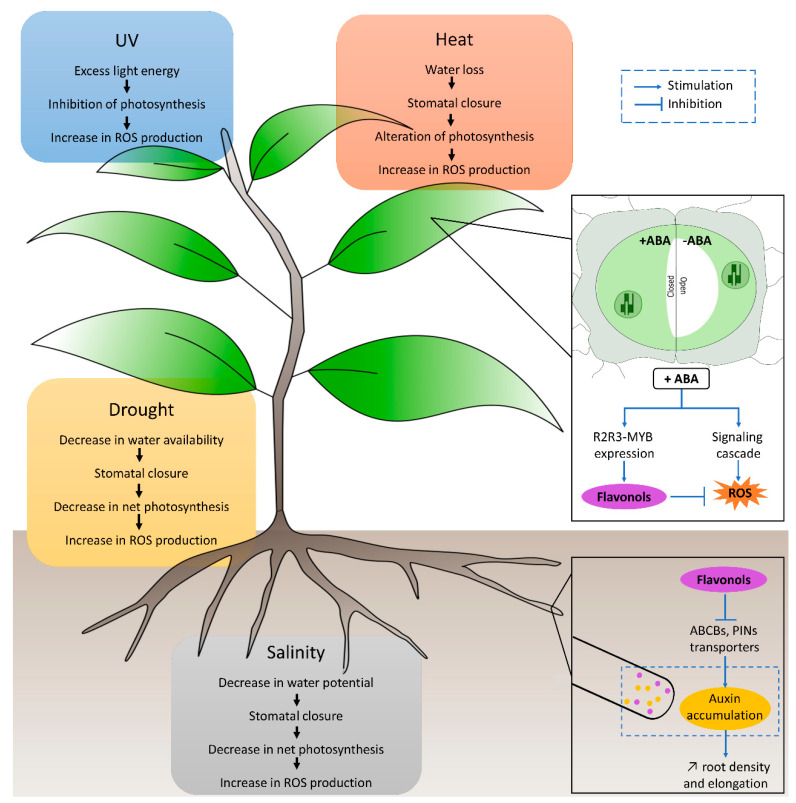
Plant responses to abiotic stress. The colored boxes summarize the four abiotic stresses referred to in this review and their main consequences for plant physiology. Flavonols’ role as ROS scavengers and their interaction with the phytohormones ABA in leaves and auxin in roots is shown in black boxes. In leaves, the opening of stomata is allowed by the binding of ABA to membrane receptors resulting in an efflux of ions and therefore an efflux of water leading to stomata closure. ABA act on the R2R3-MYB gene by enhancing its expression thus stimulating flavonol biosynthesis. It also triggers a signaling cascade leading to ROS production. In roots, flavonols inhibit auxin transport leading to auxin accumulation and root elongation.

Phytohormones allow coordination of the biosynthesis of defense compounds such as flavonoids during abiotic stress response [169] (Figure 3). The main phytohormones involved in abiotic stress responses are auxins, abscisic acid (ABA), salicylic acid (SA), jasmonic acid (JA) and ethylene (ET) [170]. There is clear evidence showing that flavonoids, as modulators of the ROS-signaling cascade, modify in turn, the phytohormone signals such as auxins and ABA [21,35].

Auxins are well-known phytohormones involved in developmental processes like growth elongation, root formation and plant tropisms in response to gravity (gravitropism) or light (phototropism) [171]. An in vitro study of Jacobs et al. [172] showed that flavonols (e.g., quercetin and kaempferol) compete with a synthetic auxin transporter inhibitor known as naphthylphthalamic acid (NPA) and can perturb auxin transport in a variety of plant tissues. Later, flavonoids’ role as negative regulators of auxin transport was proven in vivo in *Arabidopsis thaliana* mutants [173,174,175]. In vivo experiments on *Arabidopsis thaliana* shoots showed that a specific flavonol bis-glycoside (i.e., kaempferol 3-*O*-rhamnoside-7-*O*-rhamnoside) acted as an endogenous polar auxin transport inhibitor thus reducing plant stature [176]. The mechanism whereby flavonols regulate auxin transport has been explored in many studies and it includes several processes. For example, quercetin reduced the auxin transport capacity of ATP-binding cassette type B (ABCB) families [21]. Furthermore, flavonols can regulate PIN function, which are a protein family of auxin efflux transporters [35,177] (Figure 3). In addition, flavonols might affect auxin transport by changing the level of ROS which have been reported to modulate polar auxin transport (i.e., polar auxin transport is altered in plants with ROS accumulation) [178]. On the other hand, auxins control flavonol biosynthesis. Auxins control WRKY23 and MYB12 transcription factors thus increasing the accumulation of flavonols and proper root growth and development [179,180]. By regulating auxin flows, flavonols play a key role in responses to abiotic stresses because they influence auxin distribution and thus take part in the control of plant organ development in response to water deficit, salinity or other stress factors. For example, auxin’s accumulation positively modulates root architecture especially the lateral root number [181].

Various studies have found that flavonols played a role in UV acclimation by regulating auxin movement and catabolism thus leading to UV-induced morphogenic responses refs. [27,56,150,182]. Moreover, better tolerance to stress may also be due to the interaction of many other hormones such as abscisic acid (ABA) with auxins resulting in the maintenance of root growth [183].

ABA is a phytohormone considered as a plant stress hormone because it plays an important role in integrating various stress signals and controlling downstream stress responses [184]. In abiotic stress, ABA plays a crucial role by regulating various physiological processes such as stomatal closure, dormancy, germination, vegetative growth and modulation of root architecture, thus conferring adaptation to drought, salt and other osmotic stresses [185]. In plants, there is an ABA flavonol relationship in which ABA regulates flavonol biosynthesis and flavonols regulate the ABA-signaling network as well [186]. At the genetic level, ABA signaling regulates the expression of the R2R3-MYB gene family which are highly responsive to light irradiance and redox-controlled [187]. MYB genes are also known to be the main transcriptional regulators of the flavonoid biosynthetic pathway genes [68] which highlights the link between the ABA signaling pathway and flavonoid biosynthesis. Although the regulation of flavonol biosynthesis by the ABA signaling pathway remains poorly documented, there is recent evidence showing that flavonols, especially quercetin derivates, may regulate the ABA signaling pathway [188]. This later study is based on the fact that in guard cells, ABA induces a signaling cascade including the synthesis of ROS thus regulating stomatal closure [189]. However, in response to osmotic stress, ROS signaling must be rapid, but it also requires ROS scavenging by flavonols to limit cell damage. Indeed, it showed that quercetin accumulated in stomata guard cells was related to the decrease in ROS (i.e., H_2_O_2_) which are required to close stomata in response to ABA [190]. These signaling cascades involving complex interaction of ABA-induced ROS and flavonols is a crucial mechanism to understand the control of stomatal aperture, especially in stress conditions such as drought (Figure 3).

## 7. Conclusions

Although the precise role of polyphenols in plants remains complex to evaluate (because of the signaling crosstalk between their production and stress response) there is abundant evidence to support that flavonoids, especially flavonols, confer protection and indirectly regulate plant growth under abiotic stress. Enhancement of the flavonoid metabolism and eventually their production under climatic stress conditions can thereby be interpreted as an improvement of the chemical defense system in the plant. While flavonoid-related research studies have mostly used experimental designs integrating a single stress under controlled conditions, we suggest future research directions should consider field experiments where the response of the flavonoid metabolism is studied under a combination of abiotic stress, such as drought and warming in the Mediterranean ecosystems since such a scenario, rather than drought alone, will occur frequently in this region. Since these unprecedently rapid changes will probably affect Mediterranean terrestrial ecosystems dramatically, this type of study is necessary to anticipate the degree of protection chemical defenses may confer to Mediterranean species. To face such conditions, plants rich in PSM such as flavonoids with antioxidant functions will likely present competitive advantages over species with little investment in these defenses.

## Figures and Tables

**Table 1:** Comparative physico-chemical properties of some of the main flavonols, flavones and flavanones. The higher the number of hydroxyl groups, the higher the antioxidant activity. An exception is made for quercetin for which the higher antioxidant activity is due to the *ortho*-dihydroxy structure in the B ring (see Figure 1).

Flavonoid Class	Compound Subclass	Number of Hydroxyl Groups	TEAC ^(3)^ Value (mM)		Maximum Absorption Wavelength (nm) ^(4)^
			^(1)^	^(2)^	
Flavonols	Kaempferol	4	1.34 ± 0.08	1.98 ± 0.13	367
Flavonols	Quercetin	5	4.7 ± 0.1	4.30 ± 0.16	371
Flavonols	Myricetin	6	3.1 ± 0.30	2.45 ± 0.35	374
Flavones	Chrysin	2	1.43 ± 0.07	0.98 ± 0.04	313
Flavones	Apigenin	3	1.45 ± 0.08	1.04 ± 0.06	337
Flavanones	Naringenin	3	1.53 ± 0.05	0.59 ± 0.08	289
Flavanonols	Taxifolin	5	1.9 ± 0.03	2.43 ± 0.12	290

^(1)^ Data extracted from Rice-Evans et al. (1996) [46]; ^(2)^ Data extracted from Melidou et al. (2005) [48]; ^(3)^ TEAC (Trolox equivalent antioxidant activity) is defined as the concentration of Trolox solution with equivalent antioxidant potential to a 1 mM concentration of the compound under investigation.; ^(4)^ Maximum absorption wavelength extracted from Rice-Evans et al. [46] and measured by spectroscopy.

**Table 2:** Increasing of flavonol concentration under different abiotic stresses reported in literature for different species and experimental conditions. All studies are performed on harvested leaves or roots.

Abiotic Stress	Flavonol Type	Species	Plant Organ	Tissue Localization	Growth Conditions	Measurement Technique	Conclusion/Function	References
Drought	Kaempferol, quercetin	*Arabidopsis thaliana*	Not specified (all plant)	Not studied	Growth chamber	LC-PDA-MS	Scavenging radical activity (Quercetin 3-*O*-glucoside and kaempferol 3-*O*-glucoside). Quercetins had a higher antioxidant activity than kaempferols.	[105]
Drought	Myricetin, kaempferol	*Populus* spp.	Leaves and root	Not studied	Growth chamber	HPLC-PDA	Antioxidant capacity.	[106]
Drought	Kaempferol, quercetin	*Trifolium repens* L.	Leaves	Not studied	Field conditions	HPLC	Under drought stress, kaempferol glycosides accumulation was related to reduced senescence and to less pronounced decreases in shoot dry weight.	[107]
Drought and UV radiation	Quercetin	*Fraxinus ornus*	Leaves	Mesophyll (in the vacuoles of cells)	Grown outdoors in an experimental plot	Confocal microscope for flavonol localization. HPLC–MS for quantification.	Increase in quercetin 3-*O*-glucoside in severe drought and excess light stresses. Potential function as H_2_O_2_ scavenger.	[38]
UV radiation	Kaempferol	*Picea abies*	Needles	Not studied	Field cabinet experiments	RP-HPLC	Potentially UV-B screening.	[108]
UV radiation	Kaempferol, quercetin	*Arabidopsis thaliana*	Leaves	Not studied	Growth chamber	UPLC-TQD	Antioxidant activity.	[109]
UV radiation	Kaempferol, quercetin	*Arabidopsis thaliana*	Leaves	Not studied	Growth chamber	UPLC-MS	Accumulation of specific flavonol glycosides, i.e., kaempferol and quercetin di- and triglycosides (rhamnosylated) in response to UV-radiation.	[110]
UV radiation	Myricetin and quercetin	*Cistus incanus* L.	Leaves	Not studied	Field conditions	HPLC–DAD	Major light-induced increases observed for myricetin and quercetin derivatives.	[111]
Low temperature	Quercetin, kaempferol and rhamnetin	*Quercus ilex* L.	Leaves	Not studied	Field conditions (forest)	HPLC–MS/MS	High amount of flavonol-hexosides detected in winter. They could contribute to photoprotection.	[112]
Heat and drought	Kaempferol, quercetin	*Citrus* spp. (Cleopatra and Carrizo)	Leaves	Not studied	Greenhouses	UPLC/ESI-QTOF-MS	Combination of heat and drought favours accumulation of kaempferol and quercetin derivatives in poorly-drought tolerant species.	[113]
Heat and salinity	Kaempferol, quercetin	*Solanum lycopersicon* L.	Leaves	Not studied	In vitro (using aerated hydroponic systems containing a modified Hoagland solution)	UHPLC/QTOF-MS	Accumulation of kaempferol and quercetin derivatives leads to lower oxidative damage when plant grow under concomitant heat and salt stress.	[114]
Salinity and UV-radiation	Quercetin	*Ligustrum vulgare*	Leaves	Epidermal, boundary of epidermal and adaxial palisade, and in the palisade parenchyma cell layers	Greenhouses	Epifluorescence microscope and Confocal Laser Scanning Microscope (CLSM) for flavonoids localization. HPLC for quantification.	Increase in quercetin 3-*O*-glycoside in response to UV-radiation and salinity stress (NaCl). Potential role as antioxidant and photoprotection.	[115]
Salinity	Kaempferol, quercetin	*Casuarina glauca*	Nodules, roots and branchets	Not studied	In vitro (using Broughton and Dillworth’s medium)	LC-HRMS	Kaempferol and quercetin derivatives accumulate in case of severe salt stress and play a key role in protection against oxidative damage.	[116]
Salinity	Kaempferol, quercetin	*Apocynum venetum* L.	Leaves	Not studied	Plant culture room	HPLC	Kaempferol and quercetin accumulation under salt stress.	[117]

Abbreviations: HPLC: High-performance liquid chromatography; HPLC–DAD: High-performance liquid chromatography-diode array detection; HPLC–MS: High-performance liquid chromatography-mass spectrometry; HPLC-PDA: High-performance liquid chromatography–photodiode array detection; LC-HRM: Liquid chromatography-high resolution metabolomics; LC-PDA-MS: Liquid chromatography-photodiode-array-mass spectrometry; RP-HPLC: Reverse phase-high performance liquid chromatography; UHPLC/QTOF-MS: Ultra-high performance liquid chromatography-quadrupole time-of-flight mass spectrometry. UPLC/ESI-QTOF-MS: Ultra performance liquid chromatography/Electrospray-ionization- quadrupole time-of-flight mass spectrometry; UPLC-MS: Ultra Performance Liquid Chromatography-mass spectrometry; UPLC-TQD: Ultra Performance Liquid Chromatography-tandem Quadrupol.

## Data Availability

Not applicable.
